# Primary care is the ideal setting to promote COVID‐19 vaccination for children

**DOI:** 10.5694/mja2.51769

**Published:** 2022-11-20

**Authors:** Katelyn Barnes, Sally Hall Dykgraaf, Lucas de Toca, Michael Wright, Michael Kidd

**Affiliations:** ^1^ Australian Government Department of Health and Aged Care Canberra ACT; ^2^ ACT Health Directorate Canberra ACT; ^3^ Rural Clinical School Australian National University Canberra ACT; ^4^ University of Melbourne Melbourne VIC; ^5^ Centre for Health Economics Research and Evaluation University of Technology Sydney Sydney NSW; ^6^ Woollahra Doctors Sydney NSW

**Keywords:** COVID‐19, Vaccination, Primary care, Child health

Strategies to increase and sustain the COVID‐19 vaccination rate among Australian children are needed

The coronavirus disease 2019 (COVID‐19) pandemic has been labelled a “generation‐defining disruption”, particularly for children.^1^ Risk of severe acute COVID‐19 for children has been low compared with adults;^2^ particularly in Australia where comprehensive public health measures have mitigated viral spread ahead of high vaccination rates.^3^ However, COVID‐19 has also had broader unintentional impacts on children's development and mental health via social isolation, school disruption, and increased worry or fear.^4^, ^5^ Vaccination against COVID‐19 is an important strategy to minimise disease impact and reduce ongoing disruptions for children.^6^ Comirnaty (Pfizer–BioNTech) and Spikevax (Moderna) have been approved for paediatric use in Australia; both have been found to be effective for children, with rare risk of severe acute side effects.^7^, ^8^, ^9^ General practice, which delivers about 83% of recommended childhood vaccinations in Australia, has been shown to be an ideal setting to also promote childhood COVID‐19 vaccination.^10^

The population of Australia is highly vaccinated against COVID‐19, with > 95% of adults (≥ 16 years of age) and 90% of all Australians fully vaccinated.^3^, ^11^ Australia's phased approach ensured availability and access to COVID‐19 vaccines, with priority access provided to the most vulnerable members of the population and high risk workers.^12^ Subsequent eligibility was staged, with healthy adults and older adolescents eligible from late August 2021, adolescents aged 12–15 years from September 2021, and children aged 5–11 years from January 2022. In August 2022, vulnerable children aged 0.5–4 years were approved for COVID‐19 vaccination.^9^, ^12^ COVID‐19 vaccination was available in 2021 through accredited general practices and community controlled health organisations, state and territory vaccination hubs, as well as specially implemented Commonwealth vaccination clinics (led by general practitioners).^12^ Vaccination sites were expanded in late 2021 to also include community pharmacies.^13^

Uptake of vaccines in Australia has been fast, with more than 75% of healthy adults and adolescents receiving two doses within 5 months of becoming eligible.^3^ COVID‐19 vaccination rates for younger children initially mirrored that of adults and adolescents, plateauing in March 2022, with 51% of Australian children aged 5–11 years receiving a first dose at the time of writing (October 2022).^3^, ^10^ Although the reasons for plateauing are unclear, increasing paediatric infection rates since early 2022, mostly associated with mild COVID‐19, may have reduced parental perceived urgency or need for vaccination.

Consistent with international experience, the proportion of Australian children vaccinated against COVID‐19 is much lower than for longstanding scheduled childhood immunisations, with > 95% of all Australian children (≥ 5 years of age) vaccinated against diphtheria, tetanus, pertussis, and poliomyelitis.^14^ However, the COVID‐19 vaccination rate among Australian children (≥ 5 years of age) is greater than the proportion vaccinated against influenza (approximately 30% in 2020, up 3% from 2019), suggesting COVID‐19 vaccine promotion has rapidly gained traction despite challenges.^14^ Although the high proportion of Australian adults and adolescents vaccinated against COVID‐19 will provide some population level protection from infection, greater vaccine coverage among Australian children is important to reduce the potential for COVID‐19 related disruptions in child development.^4^, ^5^ Accordingly, strategies are needed to counter relative delays in COVID‐19 vaccine uptake for children.

Reasons underpinning COVID‐19 vaccine delay among children are complex and differ from those reported in other populations or for other vaccines.^15^, ^16^ Broadly, factors contributing to COVID‐19 vaccine delay in children include caregiver concerns regarding the safety and effectiveness of vaccines, or misunderstanding of the risks of infection and the benefits of vaccination for children.^16^, ^17^ For high risk, culturally and linguistically diverse groups, communication challenges and changing information may be problematic.^10^, ^18^ For socio‐economically disadvantaged groups, access to vaccination and follow‐up for subsequent doses has proved challenging.^10^ Strategies to support COVID‐19 vaccine acceptance and uptake must ensure vaccine access, and promote factual, easily understood communication from trusted sources about infection risk, isolation and vaccine side effects at individual, family and community levels.^15^, ^16^, ^17^, ^18^ Solutions to the challenge of childhood vaccination delay are multifactorial, and will require layered interventions at local, state/territory and national levels, similar to recently reported experiences with influenza vaccines.^10^, ^19^

General practice is the ideal setting to promote childhood COVID‐19 vaccine acceptance and uptake in Australia. To date (October 2022), Australian primary care settings have delivered over 60% of COVID‐19 vaccines administered nationally, with 6884 sites located in general practices (*n* = 3896), community pharmacies (*n* = 2776), Aboriginal Community Controlled Health Services (*n* = 136) and Commonwealth vaccination clinics (*n* = 76) led by GPs.^3^ About 50% of Australia's total 63 million vaccine doses were administered through general practices.^3^ GPs and general practice nurses are trusted vaccine providers and information sources, who have established relationships with children and their family members.^16^, ^17^ They are trained in techniques for difficult vaccinations, such as needle phobia or sensory sensitivities, have the capacity and experience to manage complications, and can coordinate care and referral for patients who require increased support or specialised interventions.^20^, ^21^ To support this experienced general practice workforce in effectively delivering and coordinating childhood vaccinations, discrete strategies may still be needed to address COVID‐19 specific vaccine delay among caregivers and to ensure vaccine access for children.

## Access‐related COVID‐19 vaccination delays

Access to COVID‐19 vaccination appointments that meet the needs of children and their families may improve uptake. In 2021, Australian parents reported willingness to have their children vaccinated by their usual GP; however, access is limited by appointment availability outside of work and school.^17^ Rapid scale‐up of paediatric vaccination for COVID‐19 may be constrained because of the practical complexities of vaccinating children, which takes extra time and space as well as additional parental and clinical support compared with adult vaccination.^22^, ^23^ While general practice may be the preferred setting to deliver childhood COVID‐19 vaccinations, access to appointments outside of usual hours may allow more patients to attend,^24^ when clinics may be less busy. Tailoring access for the local community has been shown to be helpful, including general practices partnering with community leaders and using community spaces, such as schools, parks and other child friendly areas to deliver vaccines for children at times and places that are convenient for patients.^10^ Community outreach programs may be particularly effective for socio‐economically disadvantaged groups.^10^ However, out‐of‐office appointments may not always be sustainable due to increased clinical and administrative staffing costs,^25^ and complex logistics for offsite medical procedures. Community pharmacies provide a quick, convenient delivery setting for non‐complex vaccination and have been important delivery sites for COVID‐19 vaccination in Australia. They may have an important adjunct role in broadening access for uncomplicated COVID‐19 vaccination in children.

Effective solutions previously implemented to increase influenza vaccination could include co‐administration of the COVID‐19 vaccines alongside other accepted childhood vaccinations.^19^ This could offer a more sustainable strategy that might improve awareness of timing and normalise childhood COVID‐19 vaccines by providing them in the context of other accepted vaccines.^14^, ^19^, ^26^ Owing to an already large role in both COVID‐19 specific and all other childhood vaccinations, general practice will be key in implementing short and long term strategies to increase COVID‐19 vaccine access for children and families.

## Delays related to caregiver COVID‐19 vaccine hesitancy

Vaccine hesitancy caused by concern or misunderstanding of COVID‐19 risks and the benefits of vaccination may be best addressed in general practice. GPs and general practice nurses are trusted sources of vaccine information, with access to child and family medical histories and existing relationships that can support tailored risk–benefit discussions with parents and caregivers.^16^, ^17^, ^26^ COVID‐19 vaccine counselling has been common in Australian general practice since vaccines became available, and is often opportunistic or iterative, with patients raising COVID‐19 vaccine concerns as part of another medical consultation.^23^ As such, GPs are ideally placed to provide counselling at a time when patients are receptive, or incrementally over time as part of the ongoing holistic care of paediatric patients. GPs and general practice nurses already use effective counselling strategies to modify patient behaviour, and can apply these to COVID‐19 vaccine counselling.^27^, ^28^, ^29^ For example, effective and efficient COVID‐19 vaccine counselling should include motivational interviewing,^27^ presumptive language,^28^ and guiding patients through COVID‐19 vaccine specific shared decision making tools.^29^ Searching practice databases may allow case finding of children who remain unvaccinated, and permit GPs and general practice nurses to reach out to patients to provide vaccine counselling and explore potential vaccine hesitancy as a trusted and familiar provider. Overall, GPs and general practice nurses are experienced in vaccine counselling and can effectively increase COVID‐19 vaccine acceptance and uptake by leveraging existing relationships with patients, and using available clinical tools to support decision making.

Broader strategies to sustainably reduce COVID‐19 vaccine hesitancy among caregivers may include primary care sites partnering with community leaders and local media (when possible) and even social media (when relevant) to support tailored but consistent messaging about the importance, safety and effectiveness of COVID‐19 vaccines alongside individual and community benefits of being vaccinated.^30^ Messaging should be clear and presented in multiple formats and languages. Partnering with community leaders will help to shape clear and meaningful messages and inspire trust, particularly for culturally and linguistically diverse groups.^18^ Consistent messaging to promote COVID‐19 vaccine acceptance should be provided in all health settings (general practice, community pharmacy, and hospitals) as well as general community settings (schools and community centres).

Primary care has an important role in supporting childhood COVID‐19 vaccine acceptance and access. General practice is a key setting in which trusted relationships with patients, families and the community can be leveraged to increase and sustain COVID‐19 vaccine acceptance and uptake among Australian children.

## Open access

Open access publishing facilitated by Australian National University, as part of the Wiley ‐ Australian National University agreement via the Council of Australian University Librarians.

## Competing interests

No relevant disclosures.

## Provenance

Not commissioned; externally peer reviewed.
